# Correlation between Maternal Vitamin D and Thyroid Function in Pregnancy with Maternal and Neonatal Outcomes: A Cross-Sectional Study

**DOI:** 10.1155/2022/6295775

**Published:** 2022-01-29

**Authors:** Salma Ahi, Mohsen Adelpour, Iman Fereydooni, Naser Hatami

**Affiliations:** ^1^Research Center for Noncommunicable Diseases, Jahrom University of Medical Sciences, Jahrom, Iran; ^2^Student Research Committee, Jahrom University of Medical Sciences, Jahrom, Iran

## Abstract

**Background:**

The aim of this study was to evaluate the prevalence of vitamin D deficiency in pregnant women to investigate the relationship between vitamin D level and thyroid function.

**Methods:**

In this cross-sectional descriptive study, a total number of 66 patients during the three trimesters of pregnancy were investigated; 22 pregnant women were studied in each trimester of pregnancy. We evaluated thyroid function tests and thyroid autoantibodies (TPOAb and TGAb), as well as the serum level of 25OHD, to determine the relationship between vitamin D level and autoimmune or non-autoimmune thyroid disease in pregnancy.

**Results:**

Pearson's correlation in all subjects showed that vitamin D levels did not have a significant relationship with maternal age. Only in the third trimester, there was a significant difference in maternal age based on their vitamin D status. There was no significant difference between the trimesters of pregnancy and vitamin D status (*P* > 0.05). Also, there were no significant differences between serum levels of vitamin D within three trimesters. Examination of thyroid function tests during pregnancy in relation to vitamin D showed that there was no significant Spearman's correlation between thyroid function status and serum vitamin D level (*P* > 0.05). There was no significant difference in the mean level of serum 25OH vitamin D in each subgroup of thyroid status (*P* > 0.05). Regarding the pregnancy outcomes, two newborns were admitted to NICU, meconium aspiration was in one case, and IUFD in another case led to pregnancy termination. These four cases were related to the maternal history of hypothyroidism.

**Conclusion:**

There was no significant relationship between vitamin D and pregnancy trimester. The serum level of vitamin D had no particular effect on the outcome of pregnancy and the thyroid gland function.

## 1. Background

Hormonal and multiple metabolic changes during pregnancy lead to complex effects on the maternal thyroid function [1]. Pregnancy increases stimulation and synthesis of steroid hormones, and it also increases the thyroxine-binding globulin's (TBG) degradation, TBG levels, total T3, and total T4. Studies have shown that TBG levels double in the 16th–20th week of pregnancy [1]. The level of thyroxin (T4) and triiodothyronine (T3) is regulated by TSH secreted from the pituitary [2, 3]. A large percentage of thyroid hormones in the bloodstream (more than 99%) are bound to the carrier proteins. Most of the thyroid hormones are transmitted by TBG and a lesser extent by transthyretin and albumin. Binding with these proteins prevents the hormone from entering the cell and provides its effect. [4]. So, thyroid disease is the second most common endocrine disorder affecting women of childbearing age [5, 6]. Recently, the evaluations of thyroid function showed that thyroid function tests interpretation depends on the stage of pregnancy [7]. Multiple hormonal and metabolic changes during each pregnancy trimester lead to complex effects on the mother's thyroid function [8]. There is a high prevalence of thyroid diseases in women of reproductive age, including chronic thyroiditis, thyroid dysfunctions, Hashimoto thyroiditis, Graves' disease, etc., and it is interesting to investigate considering factors affecting the thyroid functions test [8, 9].

Thyroid disorders affect the reproduction and the consequence of pregnancy. Pregnancy hyperthyroidism is common (0.2%), but hypothyroidism is more prevalent (2.5%) and can affect the neonatal nervous system development and also increase the incidence of congenital complications [5]. Hypothyroidism is associated with fetus nervous system poor evolution [10, 11] and increases the incidence of low birth weight (LBW) [12]. Also, the incidence of spontaneous abortions in pregnant women with chronic autoimmune thyroiditis is more prevalent [13].

Recent studies have revealed that vitamin D deficiency is prevalent worldwide; about one billion people in developing countries have vitamin D deficiency [14]. The definition of vitamin D deficiency is different in studies. While the vitamin D deficiency in Asia and the Middle East is more than other parts of the world [15, 16], the prevalence of vitamin D deficiency in Iran is similar to other Middle Eastern countries, about 69% of population [17].

During pregnancy, vitamin D requirement in some situations might be increased. Reports indicated a physiological increase in serum 25 OHD levels in the second and third trimesters. Also, it was indicated that the active vitamin D metabolite increases in pregnancy and results in‏ more intestine calcium absorption and a higher third trimester level of serum total calcium [18].

Recent studies indicate vitamin D deficiency in pregnancy, especially in high-risk groups, including vegetarians, women with low sun exposure, or ethnic minorities like blacks [19–21]. Low levels of vitamin D correlate with various maternal complications such as pregnancy-induced hypertension, hypertension in diabetic mothers, gestational diabetes, recurrent abortion, preterm labor, preeclampsia, and postpartum depression in different studies [22–27]. Vitamin D receptor is an intracellular, steroid receptor expressed by multiple organs: the brain, heart, skin, glands, prostate, breast, etc. [28]. Thyroid hormones and vitamin D receptors are the same as the steroid hormone receptor, which has various genes involved in its expression, and any modification in an individual's constructor genes is prone to autoimmune diseases, including thyroid autoimmune diseases (such as Hashimoto and Graves). So, in each thyroid immune and nonimmune thyroid disease, the level of vitamin D should be in mind [29, 30].

## 2. Method

This descriptive cross-sectional study was conducted in a community of pregnant women referred to clinics of the Jahrom University of Medical Sciences during 2018–2019. This study was approved by the Research Ethical Committee of the Jahrom University of Medical Sciences (with registration code of IR.JUMS.REC.1396.036). We divide the pregnancy into three 3-month periods or trimesters, and from each trimester, 22 pregnant Caucasian women with no acute illness, metabolic bone disorders, absorption impairment, history of thyroidectomy or radioactive iodine intake, on a free diet, without supplementation, were evaluated for FT4, TSH, 25 (OH) D, and, if necessary, FT3.The levels of thyroid antibodies (TPOAb and TGAb) were also checked to identify the relationship between vitamin D and autoimmune thyroid diseases in pregnancy. The results were interpreted based on the pregnancy trimester. After determining the mean serum level of vitamin D in each trimester, patients were divided into four groups: participants with adequate levels of vitamin D or vitamin-D-sufficient pregnant women and participants with severe, moderate, and mild vitamin D deficiency.

Blood samples were taken from all participants after at least 8 h of fasting. Free T3, free T4, and TSH were measured by Cobas ECLIAs (Roche Diagnostics GmbH, Mannheim, Germany). Thyroid peroxidase antibody (TPOAb) was determined by using chemiluminescent IMMULITE 2000 XPi (Siemens, Eschborn, Germany). Thyroid globulin antibody (TGAb) levels were analyzed by enzyme-linked immunosorbent assay (ELISA kit, Diesel).Vitamin D levels were measured by LIAISON vitamin D chemiluminescence immunoassay (DiaSorin, Saluggia, Italy).

Severe vitamin D deficiency was defined as serum 25OHD levels less than or equal to 8 ng/ml; moderate vitamin D deficiency was considered to be serum 25OHD levels from 8 ng/ml to 15 ng/ml; and mild vitamin D deficiency was 15 ng/ml to less than or equal to 20 ng/ml. Vitamin D sufficiency is defined with serum 25OHD levels higher than 20 ng/ml [31]. Pregnancy complications such as preeclampsia, preterm delivery, postpartum abnormal bleeding, or low Apgar score were recorded based on the patient's report and medical records.

In order to compare the quantitative continuous variables, ANOVA for parametric data and Mann–Whitney *U* and Kruskal–Wallis tests for nonparametric data were used. The chi-square test was used to compare discrete data among different groups. A *P* value of less than 0.05 was considered statistically significant. SPSS v.19 was used for statistical analysis.

## 3. Results

In our study, a total number of 66 patients during the three trimesters of pregnancy were investigated for thyroid function. 22 mothers in the first trimester, 22 in the second, and 22 in the third trimester were examined. The mean age of participants was 28.34 ± 4.38 years. The mean age of individuals surveyed in each trimester is presented in Table 1. There was no significant difference between the age of participants in the three trimesters (*P*=0.688).

The mean serum vitamin D was 29.24 ± 15.72 ng/ml. Severe vitamin D deficiency was observed in 1 case (1.51%), moderate vitamin D deficiency in 18 pregnant women (27.27%), mild vitamin D deficiency in 11 participants (16.66%), and vitamin-D-sufficient participants were 36 cases (54.54%).

**Figure 1:**
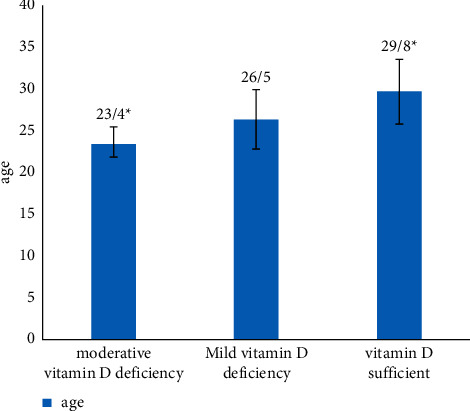
Age of participants based on the vitamin D status in the 3^rd^ trimester. Mean age of participants based on serum vitamin D status: severe vitamin D deficiency, serum 25OHD levels ≤ 8 ng/ml; moderate vitamin D deficiency, serum 25OHD levels from 8 ng/ml to 15 ng/ml; and mild vitamin D deficiency, serum 25OHD levels from 15 ng/ml to ≤20 ng/ml. ^∗^P value less than 0.05 is considered significant.

To assess the relationship between age and vitamin D, Pearson's correlation test was carried out, which indicated that vitamin D levels did not have a significant correlation with maternal age (*P*=0.071). After splitting the data based on trimesters, only in the third trimester, there was a significant difference between the ages of mothers based on their vitamin D status. The mean age of patients with normal vitamin D (29.8 ± 3.87) was higher than the mean age of vitamin-D-deficient patients (23.4 ± 1.51), as shown in Figure 1 (*P*=0.007).

The status and level of vitamin D in each trimester of pregnancy were examined and are presented in Table 1. The mean levels of serum 25OH vitamin D in pregnant women based on pregnancy trimester are presented in Table 1. The high-risk group was determined based on vitamin D deficiency, and there was no significant difference between the three trimesters of pregnancy and vitamin D status in the chi-square test (*P*=0.573). Also, there were no significant differences between the levels of serum 25OHD in each trimester compared with the others (*P*=0.381).

The results of TSH, T3, and T4 levels based on trimesters are shown in Table 1. For the nonparametric tests, these results are reported as median ± IQR. No differences were observed between three trimesters for TSH (*P* > 0.05). Also, the interpretation of thyroid function tests for the definition as thyroid disorders was also reported in Table 1, in which no significant differences were observed in the distribution of euthyroid, hypothyroid, and hyperthyroid individuals between each pregnancy trimester (*P*=0.305) and also no statistical differences were observed based on thyroid autoimmunity (see Table 2; *P*=0.24 and *P*=0.46 for TPOAb and TGAb, respectively). No significant difference was observed in Ab type and levels.

After splitting the data based on the trimesters, examination of thyroid function tests during pregnancy in relation to vitamin D showed that there was no significant Spearman's correlation between thyroid function status and serum vitamin D level (*P* > 0.05).

Mother's thyroid function tests are summarized in Table 2. There was no significant difference in the mean level of serum 25OH vitamin D in each subgroup of thyroid status (*P* > 0.05).

According to pregnancy outcomes, two newborns were admitted to the neonatal intensive care unit (NICU) and a case of meconium aspiration and a case of intrauterine fetal death (IUFD) were observed. These four cases were related to maternal history of hypothyroidism. There was no relationship between delivery type and vitamin D status (*P*=0.398; see Table 3) after exclusion of volunteered (not medically indicated) cesarean sections. There was no significant difference in mean serum 25OHD levels between pregnancy termination by cesarean section and normal delivery (*P*=0.811).

The relationship between vitamin D status and newborn outcomes studied, such as height, weight, head circumference, bilirubin, Apgar score, and gestational age, were analyzed by ANOVA. There were no significant differences in neonatal outcomes based on maternal vitamin D status during pregnancy (*P* > 0.05).

## 4. Discussion

The aim of this study was to investigate the relationship between serum vitamin D level and thyroid function tests in pregnant women referring to the Jahrom University of Medical Sciences clinics during the years 2018–2019. In this study, 45.44% of studied pregnant women had vitamin D deficiency. In a meta-analysis study in Iran, the prevalence of vitamin D deficiency among pregnant women was found to be 68.8% based on a cut-off point of 20 ng/ml [32]. This, similar to our study, was a very high prevalence.

According to the importance of first trimester in fetal organogenesis, attention to vitamin D status is crucial. Our study showed that mothers of older age had a higher level of vitamin D in the third trimester; it seems to be related to the higher level of education and better socioeconomic level of older mothers in this study. Similar to our study, Ates et al. stated no relationship between maternal age and vitamin D levels in the first trimester [33].

Different factors such as wearing hijab, residence place, and lower maternal age affect this process [34]. In our study, in the third trimester, women with higher age had higher vitamin D levels. In this study, there was no significant difference in serum levels of vitamin D comparing trimesters. Also, there was no significant difference between the trimesters of pregnancy and the prevalence of vitamin D deficiency.

Azizi et al. concluded that, during pregnancy, significant changes in the regulation of thyroid function in healthy women are observed. Increasing estrogen increases total TBG and T4. Increased hCG stimulates the thyroid gland, so TSH concentration decreases. Thyroxine metabolism is accelerated, and urinary iodine excretion is increased. In areas with iodine deficiency, there is a greater reduction in thyroxin levels and goiter during pregnancy. The prevalence of thyroid dysfunction in pregnancy was about 2 to 3%, but subclinical hypothyroidism was about 10%. Hyperthyroidism intensifies in the first trimester. Then, during the second and third trimester, the relative recovery is increased and rebounded after delivery. Infant and fetal hyperlipidemia can be caused by the passage of stimulant antibodies from the mother's TSH receptor from the placenta, causing tachycardia, accelerating bone growth, and delaying intrauterine growth. Failure to pay attention to thyroid disorders during pregnancy causes irreversible lesions in pregnancy outcomes and physical and mental development of fetuses and newborns. Therefore, the diagnosis, evaluation, and proper care of thyroid activity and its diseases during pregnancy are very important [35].

Our study demonstrated the incidence of thyroid disease was not related to vitamin D levels. There was also no difference in the incidence of thyroid diseases in pregnancy trimesters. The levels of TSH, T3, and T4 did not differ significantly comparing the trimesters.

Previous studies have confirmed the same findings. A study by Musa et al. showed that there is no clear relationship between thyroid function and vitamin D[25]OH in pregnant women [36]. Zhao et al. showed that there was no significant relationship between serum vitamin D levels and thyroid parameters [37]. Nizar et al. showed that there was no significant correlation between vitamin D levels and thyroid function of pregnant women in Oman and Jordan, but there was a relationship between higher concentrations (>30 ng/ml) of vitamin D and lower TSH [38]. However, Mackawy et al. showed that vitamin-D-deficient hypothyroid patients' vitamin D and serum calcium levels are significantly associated with hypothyroidism severity [39]. In our study, the severity and the course of the disease were not taken to account.

Studies in North China have shown a high prevalence of vitamin D deficiency in pregnant women who have not used supplements. According to these studies, there is no clear relationship between levels of vitamin D and thyroid parameters [37, 40].

There was no significant difference between the mean vitamin D levels among patients undergoing cesarean section and normal vaginal delivery in our study. Similar to the study of Asadi et al. , the relationship between vitamin D and type of delivery was investigated [41]. In the study of Savvidou et al., there was no relationship between vitamin D deficiency in the first trimester of pregnancy and cesarean section [42]. In the study of Brunvand et al., there was no relationship between vitamin D deficiency at delivery and type of delivery [43].

Despite our study and previous similar studies that observed no significant difference in the serum levels of vitamin D among women based on delivery types—cesarean section and normal vaginal delivery—Scholl et al. reported that vitamin D deficiency at 13 weeks of gestation was associated with an increase in cesarean delivery that was due to prolonged labor [44].

Regarding the relationship between vitamin D status and newborn outcomes such as height, weight, head circumference, bilirubin, agar score, and gestational age, there were no significant differences in neonatal outcomes based on maternal vitamin D status during pregnancy; in comparison with our study, neonatal jaundice as the most common neonatal problem was correlated with maternal vitamin D deficiency [45].

### 4.1. Limitations and Strengths

In this study, two major endocrine aspects of pregnancy—thyroid and vitamin D status—were investigated. For better evaluation, trimester-specific view and maternal and neonatal outcomes were observed, which are clearly helpful for clinical management. These study participants were all from the same area in the southwest of Iran with same race, wore hijabs, and experienced same climate; however, it is better to define clear sun exposure hours and nutritional status in future studies.

## 5. Conclusion

There was no significant relationship between vitamin D and pregnancy trimester. The serum level of vitamin D had no particular effect on the outcome of pregnancy and the thyroid gland function.

## Figures and Tables

**Table 1:** Studied population characteristics in different trimesters.

		First trimester	Second trimester	Third trimester	*P*
Age, mean ± SD, years		28.04 ± 4.31	28.04 ± 5.21	29.21 ± 4.52	0.688
Vitamin D levels, mean ± SD, ng/ml		29.21 ± 4.53	28.05 ± 5.21	28.05 ± 4.31	0.381

Vitamin D status, *n* (%), ng/ml	Severe vitamin D deficiency	1 (4.55)	0 (0)	0 (0)	0.573
	Moderate vitamin D deficiency	6 (27.27)	7 (31.81)	5 (22.72)	
	Mild vitamin D deficiency	5 (22.74)	4 (18.18)	2 (9.09)	
	Normal	10 (45.45)	11 (50)	15 (68.1)	

T3, median (IQR), mg/ml		15.38 (4.07–28.52)	12.33 (1.23–13.32)	14.39 (10.43–17.55)	0.088
T4, median (IQR), mg/ml		4.95 (4.27–5.28)	4.05(3.4–4.5)	4.25(4.03–4.46)	0.074
TSH, median (IQR), mIU/L		2.58 (0.58–3.64)	1.59 (0.57–3.13)	3.41 (1.38–5.12)	0.979

Thyroid function, *n* (%)	Hyperthyroidism	1 (4.54)	1 (4.54)	4 (18.18)	0.305
	Euthyroid	16 (72.72)	20 (90.90)	14 (63.63)	
	Subclinical hypothyroidism	4 (18.18)	0 (0)	3 (13.63)	
	Hypothyroidism	1 (4.54)	1 (4.54)	1 (4.54)	

A *P* value of less than 0.05 is considered significant. Normally distributed variables are shown as mean ± SD. Nonparametric variables are shown as median (IQR). IQR, interquartile range; *n*, number; TSH thyroid-stimulating hormone.

**Table 2:** Results of thyroid tests and vitamin D level in each group.

Subgroups	*N*	Vitamin D level (ng/ml)	TPOAb positive (*n*)	TGAb positive (*n*)
Hyperthyroidism	6	22.07 ± 11.13	3	3
Euthyroid	50	23.84 ± 12.42	18	15
Subclinical hypothyroidism	7	18.17 ± 5.94	5	2
Hypothyroidism	3	50.43 ± 51.6	2	2
*P* value	—	0.589	0.249	0.463

A *P* value of less than 0.05 is considered significant. TPOAb, thyroid autoantibody; TGAb, thyroglobulin antibody.

**Table 3:** Neonatal outcomes and maternal serum vitamin D.

Maternal serum vitamin D	C-section, *n* (%)	Neonatal weight (gr)	Height (cm)	Head circumference (cm)	Gestational age of birth (weeks)	Apgar (10 points)	Bilirubin level (mg/dl)
Severe vitamin D deficiency, *n* = 1	0 (0)	2550	47	34	36	9	6
Moderate vitamin D deficiency, *n* = 18	4 (22.2)	3323.33 ± 427.76	51.83 ± 2.5	33.81 ± 1.22	37.5 ± 1.04	9 ± 0	6.78 ± 0.73
Mild vitamin D deficiency, *n* = 11	5 (27.77)	3204.55 ± 346.74	51.18 ± 2.99	33.83 ± 1.47	36.91 ± 2.3	9 ± 0	7.45 ± 1.13
Vitamin D sufficient, *n* = 36	15 (41.66)	3160.56 ± 325.51	51.97 ± 2.95	33.17 ± 1.65	37 ± 1.37	9 ± 0	7.34 ± 1.45
*P* value	0.398	0.133	0.335	0.497	0.538	—	0.258

A *P* value of less than 0.05 is considered significant. Maternal vitamin D status based on serum vitamin D: severe vitamin D deficiency, serum 25OHD levels ≤ 8 ng/ml; moderate vitamin D deficiency, serum 25OHD levels from 8 ng/ml to 15 ng/ml; and mild vitamin D deficiency, serum 25OHD levels from 15 ng/ml to ≤20 ng/ml. Neonatal outcomes: birth weight (gram), height (cm), Apgar (10 points), gestational age (weeks), and serum bilirubin (mg/dl).

## Data Availability

There are no additional data. All data generated or analyzed during this study are included in this published article.
